# Intensified Job Demands and Cognitive Stress Symptoms: The Moderator Role of Individual Characteristics

**DOI:** 10.3389/fpsyg.2021.607172

**Published:** 2021-04-22

**Authors:** Johanna Rantanen, Pessi Lyyra, Taru Feldt, Mikko Villi, Tiina Parviainen

**Affiliations:** ^1^Department of Psychology, University of Jyväskylä, Jyväskylä, Finland; ^2^Department of Language and Communication Studies, University of Jyväskylä, Jyväskylä, Finland

**Keywords:** intensified job demands, cognitive stress symptoms, competence demands-related negative affectivity, competence demands-related positive affectivity, multitasking preference

## Abstract

Intensified job demands (IJDs) originate in the general accelerated pace of society and ever-changing working conditions, which subject workers to increasing workloads and deadlines, constant planning and decision-making about one’s job and career, and the continual learning of new professional knowledge and skills. This study investigated how individual characteristics, namely negative and positive affectivity related to competence demands, and multitasking preference moderate the association between IJDs and cognitive stress symptoms among media workers (*n* = 833; 69% female, mean age 48 years). The results show that although IJDs were associated with higher cognitive stress symptoms at work, that is, difficulties in concentration, thinking clearly, decision-making, and memory, competence demands-related negative affectivity explained the most variance in cognitive stress symptoms. In addition, IJDs were more strongly associated with cognitive stress symptoms at work in individuals with high competence demand-related negative affectivity, and low multitasking preference (moderation effects). Altogether, the present findings suggest that HR practices or workplace interventions to ease employees’ negative affectivity from increasing competence demands at work could usefully support employees’ effective cognitive functioning when confronted with IJDs.

## Introduction

Smooth information processing, that is, effective cognitive functioning, is required every day at work, especially for knowledge workers. There is, however, no guarantee nowadays that working conditions will support effective cognitive functioning. Intensified job demands (henceforth IJDs), resulting from the acceleration of working life and digitalization (Kubicek et al., 2015), may burden particularly white-collar and knowledge workers and lead to impaired task performance (Mauno et al., 2020), as well as cognitive strain when information processing capacity is overloaded (Vuori et al., 2019; Kalakoski et al., 2020). Here, we chose to study media workers because as an occupational group they represent knowledge workers in a high-speed, high-pressure, and highly digitalized working environment. By applying *the job demands-resources theory* (Demerouti et al., 2001; Bakker and Demerouti, 2017) we first investigated whether IJDs are related to cognitive stress symptoms among media workers. Second, using *the framework for studying personality in the stress process* (Bolger and Zuckerman, 1995), we examined the possible moderator role of media workers’ negative and positive affectivity related to competence demands, as well as multitasking preference, in the association between IJDs and cognitive stress symptoms. Understanding the interplay between situational and individual factors behind cognitive stress symptoms is helpful in, for example, planning research-based HR practices and workplace interventions directed at employees who work in demanding conditions.

### IJDs and Cognitive Stress Symptoms in the Context of Media Work

Kubicek et al. (2015) have introduced the concept of IJDs, which can be defined as “job characteristics which intensify and accelerate the pace of work, job- and career-related planning and decision-making and work-related learning” (Mauno et al., 2019, p. 224). The very nature of continuous *intensification* and *acceleration* of job demands differentiates IJDs from the traditional job demands, which according to Karasek (1989) refer to stress inducing job contents and duties that require considerable psychological or physical effort. Accordingly, based on the theory of social acceleration (Rosa, 2003) and on observations of the constantly increasing demands on employees (e.g., Obschonka et al., 2012), Kubicek et al. (2015) define five dimensions of IJDs.

The first and most important dimension of IJDs is *work intensification*, that is, employees’ need to work faster, multitask more, and invest more and more effort in their everyday work. The second dimension of IJDs consists of *intensified job-related planning and decision-making demands*. This dimension refers to the expectation that employees will increasingly take autonomous responsibility for their work, from the stage of goal-setting to the monitoring of the end results (Kubicek et al., 2015). The third dimension, *intensified career-related planning and decision-making demands*, refers to the view that it is up to employees to ensure that their career prospects are secured and their employability is high, both within and outside the organization. Finally, the fourth and fifth dimensions, *intensified knowledge-related learning* and *intensified skill-related learning demands*, refer to the requirement that in order to keep up with ever-changing working life and the latest developments in their field, employees have to constantly renew their expertise regarding the content (i.e., knowledge) as well as the equipment, practices, and programs (i.e., skills) of their work.

Job demands in general (Albertsen et al., 2001), and specifically from digitalization (Vuori et al., 2019), may trigger a variety of stress-related symptoms in employees at both the somatic level (e.g., increased blood pressure) and the psychological level (e.g., anxiety). Especially relevant for knowledge workers are cognitive stress symptoms, such as working memory difficulties, indifference toward work, information overload, and impaired ability to concentrate (Albertsen et al., 2010; Kalakoski et al., 2020). In the present study, cognitive stress symptoms were therefore operationalized as self-reported difficulties at work in concentrating, thinking clearly, making decisions, and remembering (see Copenhagen Psychosocial Questionnaire; Pejtersen et al., 2010).

In this study, the association between IJDs and cognitive stress symptoms was examined in the context of media work. Media work refers to planning, producing, and marketing media content, products, services, and brands within media organizations (Deuze, 2007). It is not limited to journalistic work but also includes other activities undertaken by media professionals (such as producers and art directors) aimed at advancing the success of media products and services (Malmelin and Villi, 2017). In comparison to workers in other industries, media workers have been among the first to have to adjust to digitalization in working life, with its positive and negative effects (Malmelin and Villi, 2017), and the more extensive job descriptions with their demands of multi-competences (Bakker, 2014; Deuze and Witschge, 2018). Moreover, due to digital disruption in the production and consumption of media products, media organizations have had to increasingly dismiss workers, which has led to work intensification among those who have kept their jobs (Villi et al., 2020).

Thus, by focusing on media workers, it is possible to narrow the approach to such a subset of knowledge workers who work in a context where digital technology, both in the work practices and the business environment at large, has made a disruptive impact (Achtenhagen and Raviola, 2009; Westlund et al., 2020). Media workers might well be at risk for stressors detrimental to cognitive performance (e.g., Vuori et al., 2019) as a consequence of the disruption of work practices by new communication technologies, increased demands for productivity, and the faster tempo of work. Together the above reviewed characteristics of media work and industry provide a solid foundation to investigate the association between IDJs and cognitive stress symptoms among knowledge workers.

### The Job Demands-Resources Theory and the Relation Between IJDs and Cognitive Stress Symptoms

According to the job demands-resources theory, and specifically its health-impairment process, various physical, social, and organizational job demands can be seen as antecedents for job strain (Demerouti et al., 2001; Bakker and Demerouti, 2017). Similarly, and supported by previous research, it seems evident that particularly work intensification, the core dimension of IJDs, poses the risk of reduced well-being at work, such as high job exhaustion and psychological strain (e.g., Chesley, 2014; Franke, 2015; Korunka et al., 2015). The other dimensions of IJDs have also been found to relate to high burnout, but not as strongly as work intensification (Mauno et al., 2019).

To our knowledge, the relationship between the different dimensions of IJDs and cognitive stress symptoms has not been investigated before, although there is likely to be a positive association between these factors, partly fueled by digitalization and information overload (Vuori et al., 2019; Kalakoski et al., 2020). This is the first research gap that the present study addresses. Indeed, Zeike et al. (2019) argue that digitalization in working life can induce high cognitive demands such as “choice overload,” which can be experienced by workers as anxious and troubling when they have to make complex decisions. Instead of IJDs, which emphasize continuous intensification and acceleration of job demands, more traditional job demands such as high workload, time pressures, and a lack of control have been found to associate with cognitive stress and failures at work (Albertsen et al., 2010; Elfering et al., 2011; Vuori et al., 2014). Following these findings and the job demands-resources theory (Demerouti et al., 2001; Bakker and Demerouti, 2017), we posed our first hypothesis:

Hypothesis 1: IJDs (i.e., work intensification, intensified job- and career-related planning and decision-making demands, and intensified knowledge- and skill-related learning demands) are positively related to cognitive stress symptoms.

It has also been shown that the association between work intensification and occupational well-being may be influenced by individual cognitive appraisal of the job demands (Paškvan et al., 2016). Workers have been found to be less satisfied and more exhausted with their work if they perceive job demands as a hindrance and stressor rather than a challenge (e.g., Podsakoff et al., 2007; Meyer and Hünefeld, 2018). How individuals experience the number and nature of job demands can also have wide-ranging consequences for their well-being. On the positive side, high cognitive demands at work do predict higher cognitive function and slower decline of cognitive capacity after retirement (Fisher et al., 2014). On the negative side, when these demands are perceived as stressors, they may expose the individual to early cognitive deficits (Sindi et al., 2017). It is therefore important to chart possible moderators of the relationship between IJDs and cognitive stress symptoms, and consider the factors that may explain individual differences in perceiving IJDs in a particular way.

### The Framework for Studying Personality in the Stress Process and the Relation Between IJDs and Cognitive Stress Symptoms

According to the framework for studying personality in the stress process, individual characteristics can affect the way workers react to job demands because differences in individual characteristics may explain why some workers perceive and experience the same situation as more demanding or stressful than others (Bolger and Zuckerman, 1995). Also, in cognitive stress research, the importance of considering both factors in the work environment and individual characteristics has been emphasized when investigating sources of stress (e.g., Albertsen et al., 2010). In light of the present study, this means that while some workers may excel under the changed conditions brought about by digitalization, IJDs may be detrimental to many workers’ functioning and might lead to cognitive stress symptoms. Self-esteem and a sense of coherence are shown to be crucial for maintaining performance in the face of cognitive stressors (Albertsen et al., 2001, 2010), suggesting that individual trait characteristics influence stress. Along these lines, we examined whether competence demands-related negative and positive affectivity on the one hand, and multitasking preference on the other, moderate the association between IJDs and cognitive stress symptoms.

As discussed in what follows, competence demands-related negative and positive affectivity represent broad individual differences at the level of dispositional traits, that is, at the level one of the three-tiered framework of personality (McAdams and Pals, 2006). Multitasking preference at the level two of this framework, on the other hand, represents a more specific motivational style of adapting to life’s various situations and circumstances. In the present study, we examined the relation between IJDs and cognitive stress symptoms from these two perspectives, as they signify affective and motivational elements, both of which are likely to contribute to the dynamics between an individual and his/her working environment, that is, person-job fit (e.g., Kristof-Brown et al., 2005; van Vianen, 2018). This is the second research gap that the present study addresses. In earlier literature, it has been more common to consider only a single trait or one level of the three-tiered framework of personality when investigating individual differences in occupational well-being (Mäkikangas et al., 2013).

Competence demands-related negative and positive affectivity refer to the affects that the current competence demands of media work elicited among our participants. This two-dimensional perspective on competence demands-related affects follows Watson and Tellegen’s (1985) traditional consensual structure of mood. According to it, emotional experiences and reactions can be divided into two dominant dimensions, negative and positive affect; the former refers to a temperamental tendency for negative mood states such as distress and dissatisfaction, and the latter to a temperamental tendency for positive mood states such as enthusiasm and contentment (Watson and Clark, 1992). Competence demands-related affectivity, although subject to change if competence demands change substantially, is thus likely to link with dispositional affectivity, which represents individual characteristic tendencies to react to events both inside us and outside us, and aligns with neurobiologically based sensitivity to reward (Gray, 1987; Corr, 2004). Positive affectivity is related to a behavioral activation system (BAS), and negative affectivity to a behavioral inhibition system (BIS) (Carver and White, 1994), understood as sensitivity to positive and negative outcomes, or rewards and punishments, respectively.

Multitasking preference is a characteristic adaptation style to today’s working life, which requires constant shifting of attention and division of effort between different job tasks. The concept of multitasking has its roots in polychronicity (Poposki and Oswald, 2010), which refers to involvement in more than one activity at a time (e.g., Slocombe, 1999). Although engagement in multitasking is considered problematic for cognitive functions (e.g., Vestergren and Nilsson, 2011), it is not, however, evidence of neither enjoyment nor mere acceptance of multitasking. This is why Poposki and Oswald (2010) suggest that to consider and measure polychronicity at an individual level, one must differentiate multitasking *behavior* (i.e., multitasking engagement) from multitasking *preference*. They define the latter as “a non-cognitive variable reflecting an individual’s preference for shifting attention among ongoing tasks, rather than focusing on one task until completion and then switching to another task” (Poposki and Oswald, 2010, p. 250). Hence, because in this study we were specifically interested in the individual characteristics that might mitigate or reinforce the possibly positive association between IJDs and cognitive stress symptoms, we focused precisely on multitasking *preference*, instead of multitasking behavior, as a moderator in the association between IDJs and cognitive stress symptoms.

The present study design is based on the differential reactivity model derived from Bolger and Zuckerman’s (1995) framework for studying personality in the stress process, a view that has been supported by literature review (Mäkikangas et al., 2013) and has been recently incorporated also to job demands-resources theory (Bakker and Demerouti, 2017). In the present study, the five dimensions of IJDs described above (Kubicek et al., 2015) are considered stressors in the work environment, and cognitive stress symptoms (Pejtersen et al., 2010) are considered possible stress reactions to IJDs. According to the differential reactivity model, stressor reactivity indicates the extent to which an individual is likely to express emotional or physiological reactions in a situation that is perceived as stressful (Bolger and Zuckerman, 1995). Individual characteristics (here, competence demands-related negative and positive affectivity and multitasking preference) can moderate the effects of stressful situations (here, IJDs) on psychological and physiological outcomes (here, cognitive stress symptoms).

The rationale for the moderation hypotheses of the present study lies also in the notion that it is important to consider the possible interplay between work environment and individual characteristics (e.g., Albertsen et al., 2010), in other words, person-job fit (e.g., Kristof-Brown et al., 2005; van Vianen, 2018), when investigating sources of stress. Competence demands-related negative affectivity might strengthen the association between IJDs and cognitive stress symptoms because individuals high in negative affectivity (or in neuroticism, a close construct; e.g., Watson and Clark, 1992) are inclined to focus on the worrying and threatening aspects of their work environment (e.g., Schaubroeck et al., 1992; Mäkikangas et al., 2013). They may thus detect IJDs more easily and experience these demands more adversely than others, and this may predispose them to cognitive stress symptoms. For the above reasons, our second hypothesis was:

Hypothesis 2: Among those high in competence demands-related negative affectivity, the positive relationships between IJDs (i.e., work intensification, intensified job- and career-related planning and decision-making demands, and intensified knowledge- and skill-related learning demands) and cognitive stress symptoms are stronger than among those low in competence demands-related negative affectivity.

Conversely, competence demands-related positive affectivity might weaken the association between IJDs and cognitive stress symptoms because individuals high in positive affectivity (or in extraversion, a close construct; e.g., Watson and Clark, 1992) are likely to focus on the inspiring and rewarding aspects of their work (e.g., Mäkikangas et al., 2013). Hence, despite detecting the presence of IJDs, they may experience these demands less adversely than others, perhaps as challenges rather than hindrances or stressors, which may protect them from cognitive stress symptoms. In addition, Boudrias et al. (2020) found that the well-met need for competence at work (an experience close to competence demands-related positive affectivity) buffered fully from the positive and significant relationship between role conflict (stressful situation) and turnover intention (job strain outcome). Based on these assumptions and findings, our third hypothesis was:

Hypothesis 3: Among those high in competence demands-related positive affectivity, the positive relationships between IJDs (i.e., work intensification, intensified job- and career-related planning and decision-making demands, and intensified knowledge- and skill-related learning demands) and cognitive stress symptoms are weaker than among those low in competence demands-related positive affectivity.

Finally, those high in multitasking preference might perceive IJDs as corresponding well with their willingness, or even desire, to divide their attention and effort between different job tasks, projects, or clients (e.g., Poposki and Oswald, 2010). Thus, they may experience IJDs more favorably than those who have low multitasking preference, and this may protect them from experiencing cognitive stress symptoms when faced with IJDs. Accordingly, our last hypothesis was:

Hypothesis 4: Among those high in multitasking preference, the positive relationships between IJDs (i.e., work intensification, intensified job- and career-related planning and decision-making demands, and intensified knowledge- and skill-related learning demands) and cognitive stress symptoms are weaker than among those low in multitasking preference.

## Materials and Methods

### Procedure, Ethical Consideration, and Participants

In 2019, an invitation to participate in an e-survey focusing on media workers’ working conditions and occupational well-being was sent by email to the members of the Union of Journalists in Finland (*N* = 13,652). This inquiry was first approved by the union, after which anonymous participation was strictly volunteer based on informed consents from all participants who were legally competent adults. In these circumstances, both the national and the regulations of (University of Jyväskylä) ethical committee exempt this study from an ethical review. Of those who received the email, 43% opened it, and of these, 17% completed the survey. A total of 833 respondents were included in the present study who, in addition to the main study variables, also provided information on the control variables: gender, age, education, and weekly working hours.

Of the respondents, 69% were women, and the mean age was 47.6 years (*SD* = 10.0). Of the respondents’ level of education, 22% held less than a bachelor’s degree, 29% had a bachelor’s degree or equivalent, 48% had a master’s degree or equivalent, and 1% had a higher degree. Most (81%) were employees in organizations of various sizes, 16% worked full-time as freelancers, and 3% had another work situation (student worker, being both an employee and a freelancer, etc.). The mean for weekly working hours was 39.2 (*SD* = 7.2).

### Measures

#### Intensified Job Demands

IJDs were measured with 13 items from the intensification of job demands scale (Kubicek et al., 2015; officially translated into Finnish by Mauno et al., 2019). These items covered all the five dimensions of IJDs, and the following instruction preceded the items: “In the last 5 years, have the following changes occurred in your work in the media field? If you have worked less than 5 years in this field, please evaluate the statements based on the time you have worked in this field.” Work intensification was measured with three items (e.g., “Ever more work has to be completed by fewer and fewer employees,” α = 0.75), intensified job-related planning and decision-making demands with three items (e.g., “One increasingly has to check independently whether the work goals have been reached,” α = 0.71), intensified career-related planning and decision-making demands with three items (e.g., “One’s own professional development increasingly requires to keep other alternatives open,” α = 0.74), intensified knowledge-related learning demands with two items (e.g., “One has to acquire new expertise for the job more often,” α = 0.72), and intensified skill-related learning demands with two items (e.g., “One has to use new work equipment (devices, programs, etc.) more often,” α = 0.77). Items were rated on a scale from 1 = *not at all* to 5 = *completely*.

#### Cognitive Stress Symptoms

Cognitive stress symptoms were measured with four items from the Copenhagen Psychosocial Questionnaire (Pejtersen et al., 2010). These items have been officially translated into Finnish in the SujuKE study (Finnish Institute of Occupational Health, (n. d).). Respondents were asked to rate on a scale from 1 = *not at all* to 5 = *all the time* their following experiences: “How often have you had difficulty at work (a) concentrating, (b) thinking clearly, (c) taking decisions, and (d) remembering?” (α = 0.83).

#### Competence Demands-Related Negative and Positive Affectivity

Competence demands-related negative and positive affectivity was measured with one question: “How often do the current competence demands of media work make you feel as described below?” Respondents rated their reaction to six negative (e.g., gloomy, tense, α = 0.85) and six positive (e.g., enthusiastic, calm, α = 0.87) affects on a scale from 1 = *never* to 6 = *constantly*. These adjectives derived from Warr’s (1990) measure for affective job-related well-being (validated in Finnish by Mäkikangas et al., 2007) because they work particularly well for occupational research. However, here these adjectives were not used to measure occupational well-being *per se*, but instead the negative and positive emotions media workers experienced in response to the current competence demands of media work.

#### Multitasking Preference

Multitasking preference was measured with five items from an inventory to measure individual differences in polychronicity (Poposki and Oswald, 2010). Respondents rated their reactions on a scale from 1 = *strongly disagree* to 5 = *strongly agree* to five items such as “I would rather switch back and forth between several projects than concentrate my efforts on just one” (α = 0.79).

#### Background Factors

In the analyses we controlled for gender (1 = man, 2 = woman), age (in years), education (1 = less than bachelor level, 2 = bachelor level, 3 = master’s level, 4 = higher than master’s level) and weekly working hours (including all work performed, regardless of time and place).

### Data Analysis

All analyses were conducted with the SPSS 24.0 statistical program. *First*, Pearson correlations were inspected between all the study variables. *Second*, we performed five hierarchical moderated regression analyses. In these analyses, cognitive stress symptoms were set as a dependent variable, background factors (gender, age, education, and weekly working hours) as control variables, each dimension of IJDs at a time as an independent variable, and individual characteristics (competence demands-related negative and positive affectivity and multitasking preference) as moderator variables. Background factors were entered at step 1 to control for their effects before each dimension of IJDs was entered to the model at step 2, to inspect their main effects on cognitive stress symptoms. After this, at step 3, individual characteristics were entered, and finally interaction terms (that is, standardized individual characteristic multiplied by the standardized dimension of the IJDs in question) were entered at step 4 to investigate the moderation effects.

In calculating the interaction terms, *z-*standardization for variables constituting the interaction term was used (Aiken and West, 1991). Also, if a significant moderation effect was detected, then simple slope tests (Cohen et al., 2003; Dawson, 2014) were used to evaluate whether the relationship (slope) between the particular dimension of IJDs and cognitive stress symptoms was significant at a particular value of the individual characteristic in question.

## Results

### Descriptive Results

Table 1 shows the means, standard deviations, and correlations of all the study variables. It shows that media workers experienced rather high IJDs consistently across the dimensions (all means above 3.5), whereas the mean 2.62 for cognitive stress symptoms was not particularly high. Of the background factors, higher age was related to lower experience of intensified career-related planning and decision-making demands, and a lower level of cognitive stress symptoms. Long weekly working hours were related to high IJDs (all dimensions) as well as to high competence demands-related negative affectivity.

**TABLE 1:** Means, standard deviations (SD), and correlations (*n* = 833) between study variables.

	***Mean (SD)***	**1.**	**2.**	**3.**	**4.**	**5.**	**6.**	**7.**	**8.**	**9.**	**10.**	**11.**	**12.**
1. Gender^a^	–	–											
2. Age	47.57 (9.96)	−0.08*	–										
3. Educational level^b^	2.28 (0.82)	0.23***	−0.16***	–									
4. Weekly working hours	39.19 (7.16)	–0.06	–0.01	–0.02	–								
5. WI (1–5)	3.87 (0.94)	0.01	–0.06	–0.05	0.16***	–							
6. IJP demands (1–5)	3.68 (0.88)	0.08*	0.00	–0.02	0.10**	0.49***	–						
7. ICP demands (1–5)	3.74 (0.90)	0.14**	−0.21***	0.11**	0.07*	0.34***	0.46***	–					
8. IKL demands (1–5)	4.02 (0.81)	0.05	0.07*	–0.01	0.09**	0.38***	0.37***	0.48***	–				
9. ISL demands (1–5)	4.17 (0.85)	0.02	0.09*	–0.05	0.10**	0.49***	0.30***	0.23***	0.62***	–			
10. Negative affectivity^c^ (1–6)	2.99 (0.89)	–0.03	−0.07*	0.01	0.11**	0.39***	0.22***	0.23***	0.18***	0.24***	–		
11. Positive affectivity^c^ (1–6)	3.28 (0.82)	0.05	0.01	0.03	0.00	−0.30***	−0.13***	−0.09*	–0.01	−0.16***	−0.59***	–	
12. Multitasking preference (1–5)	3.10 (0.73)	0.13***	–0.04	0.09**	0.01	−0.13***	0.06	0.05	–0.02	−0.09**	−0.19***	0.26***	–
13. Cognitive stress symptoms (1–5)	2.62 (0.74)	0.06	−0.21***	0.07*	0.06	0.21***	0.14***	0.18***	0.11**	0.12***	0.45***	−0.29***	−0.11**

Response scales in parentheses, ^*a*^1, man; 2, woman; ^*b*^1, less than bachelor’s level …; 4, higher than master’s level; ^*c*^competence demands-related affectivity; WI, work intensification; IJP, intensified job-related planning and decision-making; ICP, intensified career-related planning and decision-making; IKL, intensified knowledge-related learning; ISL, intensified skill-related learning. *p < 0.05, **p < 0.01, ***p < 0.01.

In addition, high IJDs (all dimensions) and high competence demands-related negative affectivity were related to high cognitive stress symptoms, while high competence demands-related positive affectivity and high multitasking preference related to low cognitive stress symptoms. Due to the strong correlation between intensified knowledge and skill-related learning demands (*r* = 0.62), and between competence demands-related negative and positive affectivity (*r* = −0.59) in the subsequent regression analyses, multicollinearity diagnostics were inspected by calculating variance inflation factors (VIF). These diagnostics, however, did not indicate the presence of multicollinearity (in all models VIFs < 2 for each variable). This, together with the fact that preliminary factor analysis showed that items for IDJs, cognitive stress symptoms, competence demands-related negative and positive affectivity and multitasking preference did not load on one common factor but instead their own factors, also confirmed that the regression models should be free of common method bias (Kock, 2015). When all the items were forced to load on one common factor (Harman’s single factor test), it accounted for only 21% of the total variance. According to Fuller et al. (2016), p. 3,197), the total variance should be “70% or more before substantial concern about inflated relationships would arise.” The detailed results concerning these preliminary factor analyses are available upon request from the first author.

### Hierarchical Moderated Regression Analyses

As seen in Table 2, at step 2 of the regression analyses the level of cognitive stress symptoms was explained by all the dimensions of IJDs even after controlling for the effects of background factors. Of the IJD dimensions, work intensification explained the most (4%) and intensified knowledge-related learning demands the least (1%) of the variance in cognitive stress symptoms. However, when at step 3 the individual characteristics were entered to the models, all the dimensions of IJDs lost their statistical significance in explaining the variance in cognitive stress symptoms. Individual characteristics (especially competence demands-related negative affectivity) explained 16–18% of the variance in cognitive stress symptoms. Hence, our Hypothesis 1, assuming a positive relationship between IJDs and cognitive stress symptoms, was only supported when the role of individual characteristics was not considered.

**TABLE 2:** Individual characteristics as moderators in the relation between intensified job demands and cognitive stress symptoms.

	**Model 1: IJDs = work intensification**		**Model 2: IJDs = job-related planning and decision-making demands**		**Model 3: IJDs = career-related planning and decision-making demands**		**Model 4: IJDs = knowledge-related learning demands**		**Model 5: IJDs = skill-related learning demands**	
**Variables**	**β*_*step*__4_***	**(β)**	**β*_*step*__4_***	**(β)**	**β*_*step*__4_***	**(β)**	**β*_*step*__4_***	**(β)**	**β*_*step*__4_***	**(β)**
**Step 1, Δ*R*^2^**	0.05***		0.05***		0.05***		0.05***		0.05***	
Gender^a^	0.05	(0.03)	0.05	(0.03)	0.04	(0.03)	0.05	(0.03)	0.05	(0.03)
Age	−0.16***	(−0.20***)	−0.17***	(−0.20***)	−0.15***	(−0.20***)	−0.17***	(−0.20***)	−0.17***	(−0.20***)
Educational level^b^	0.04	(0.04)	0.04	(0.04)	0.03	(0.04)	0.03	(0.04)	0.04	(0.04)
Weekly working hours	0.02	(0.07)	0.02	(0.07)	0.02	(0.07)	0.02	(0.07)	0.02	(0.07)
**Step 2, Δ*R*^2^**	0.04***		0.02***		0.02***		0.01**		0.02***	
IJDs ^c^	0.04	(0.20***)	0.06	(0.14***)	0.04	(0.13***)	0.05	(0.12**)	0.03	(0.14***)
**Step 3, Δ*R*^2^**	0.16***		0.18***		0.18***		0.18***		0.18***	
Negative affectivity^d^	0.39***	(0.40***)	0.39***	(0.40***)	0.39***	(0.40***)	0.39***	(0.40***)	0.40***	(0.40***)
Positive affectivity^d^	–0.03	(−0.04)	–0.04	(−0.04)	–0.04	(−0.04)	–0.05	(−0.05)	–0.04	(−0.04)
Multitasking preference	–0.05	(−0.04)	–0.05	(−0.04)	–0.04	(−0.04)	–0.04	(−0.04)	–0.04	(−0.04)
**Step 4, Δ*R*^2^**	0.01*		0.01*		0.01**		0.00		0.00	
IJDs^*c*^ × Negative affectivity ^d^	0.09*		0.07		0.08*		0.06		0.02	
IJDs^*c*^ × Positive affectivity ^d^	0.06		–0.01		0.03		0.04		0.04	
IJDs^*c*^ × Multitasking preference	−0.07*		–0.05		−0.08*		–0.03		–0.02	
***R*^2^ for whole model at Step 4**	0.24***		0.24***		0.24***		0.24***		0.23***	

***β_*step*__4_***, the standardized beta coefficient for each variable in the fourth and last step of the regression model; (β), the standardized beta coefficient for each variable before step 4, at the step at which the variable is entered to the regression model; ^*a*^1, man; 2, woman; ^*b*^1, less than bachelor’s level …; 4, higher than master’s level; ^*c*^IJDs, intensified job demands, and that the dimension used in each model appears in the top line, ^*d*^competence demands-related affectivity. *p ≤ 0.05, **p < 0.01, ***p < 0.01.

**FIGURE 1:**
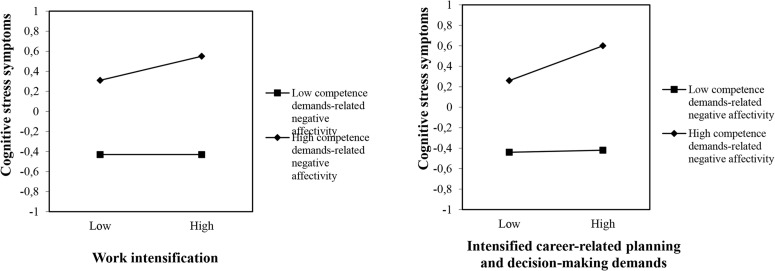
Competence demands-related negative affectivity as a moderator in the association between work intensification/intensified career-related planning and decision-making demands and cognitive stress symptoms. The scale on the *Y*-axis as well as the entries “low” and “high” refer to –1 and +1 standard deviation below and above the sample mean in the variable in question.

**FIGURE 2:**
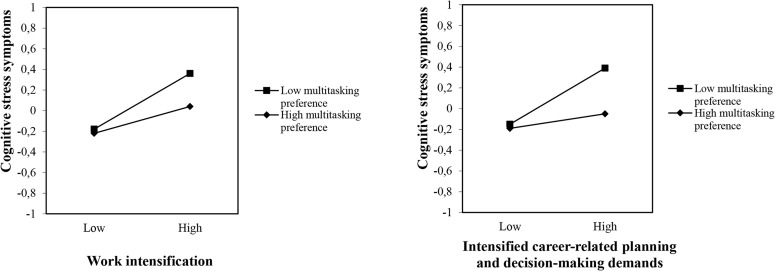
Multitasking preference as a moderator in the association between work intensification/intensified career-related planning and decision-making demands and cognitive stress symptoms. The scale on the *Y*-axis as well as the entries “low” and “high” refer to –1 and +1 standard deviation below and above the sample mean in the variable in question.

As shown in Table 2 at step 4, four moderator effects for cognitive stress symptoms were detected. The interaction terms competence demands-related negative affectivity × work intensification, multitasking preference × work intensification, competence demands-related negative affectivity × intensified career-related planning and decision-making demands, and multitasking preference × intensified career-related planning and decision-making demands were statistically significantly related to cognitive stress symptoms. These effects are illustrated in Figures 1, 2. These graphical representations of the moderator effects were derived using the standardized regression coefficients of the regression lines for respondents with high (1 *SD* above the mean) and low (1 *SD* below the mean) scores on the moderator.

As shown in Figure 1, respondents high in competence demands-related negative affectivity reported overall more cognitive stress symptoms than those low in this characteristic, especially when experiencing high work intensification and high intensified career-related planning and decision-making demands. The simple slope tests revealed that the positive associations between work intensification and cognitive stress symptoms, and between intensified career-related planning and decision-making demands and cognitive stress symptoms, were significant among those high in competence demands-related negative affectivity (β = 0.13, *p* < 0.05 and β = 0.17, *p* < 0.001, respectively), but non-existent among those low in this characteristic (β = 0.00, *p* = n.s. and β = 0.01, *p* = n.s., respectively). These findings partly support our Hypothesis 2. The assumed opposite findings for competence demands-related positive affectivity were not detected, so Hypothesis 3 was not supported.

As shown in Figure 2, for those who experienced high work intensification and intensified career-related planning and decision-making demands, respondents with high multitasking preference reported fewer cognitive stress symptoms than respondents with low multitasking preferences. For those who experienced less work intensification and fewer intensified career-related planning and decision-making demands, the preference for multitasking did not influence the reported low level of cognitive stress symptoms. Simple slope tests revealed that the positive associations between work intensification and cognitive stress symptoms, and between intensified career-related planning and decision-making demands and cognitive stress symptoms, were significant among all respondents, but were weaker among those high in multitasking preference (β = 0.13, *p* < 0.01 and β = 0.08, *p* < 0.10, respectively), and stronger among those low in multitasking preference (β = 0.26, *p* < 0.001 and β = 0.27, *p* < 0.001, respectively). These findings partly support Hypothesis 4.

## Discussion

Due to the acceleration of working life, it has been suggested that nowadays it is not just job demands but particularly *intensified* job demands (IJDs) that challenge workers’ resources and occupational well-being (Kubicek et al., 2015). IJDs induced by technological advances and digitalization are particularly prevalent among white-collar workers (e.g., Mauno et al., 2020) and may overburden knowledge workers’ information processing capacity and lead to cognitive strain at work (Vuori et al., 2019; Kalakoski et al., 2020). For these reasons, the present study aimed to extend the still incipient research concerning the relation between IJDs and occupational well-being (e.g., Korunka et al., 2015; Mauno et al., 2019). To our knowledge, cognitive stress symptoms have not been studied before in conjunction with IJDs, and investigating the moderating role of individual characteristics in the relation between IJDs as stressor and cognitive stress symptoms as a possible stress reaction to IJDs also offers new understanding of both these phenomena.

The present results showed that at first all five dimensions of IJDs associated with heightened experience of cognitive stress symptoms. Of the dimensions, work intensification explained most of the variance in the reported level of cognitive stress symptoms when background factors, particularly age (younger respondents reported more cognitive stress symptoms), were controlled for. However, the association between IJDs and cognitive stress symptoms were lower in magnitude (see Tables 1, 2) than earlier observations of the association between IJDs and job exhaustion (Korunka et al., 2015) and burnout (Mauno et al., 2019). This is somewhat surprising, because IJDs have been characterized as predominantly *cognitive* in nature (Chesley, 2014; Kubicek et al., 2015; Mauno et al., 2020), and because the media workers in this study reported generally rather high overall experience of IJDs. On the other hand, it is known that a certain amount of cognitive job demands may even enhance cognitive functioning and thus buffer the age-related decline in these functions (e.g., Fisher et al., 2014; Hussenoeder et al., 2019). The same could apply also in one’s current work, especially if an individual appraises job demands as positive (i.e., as challenges) rather than negative (i.e., as hindrances) aspects of one’s work (e.g., Podsakoff et al., 2007; Paškvan et al., 2016; Meyer and Hünefeld, 2018).

When the role of individual characteristics was considered in the associations between IJDs and cognitive stress symptoms, important findings emerged. Competence demands-related negative affectivity explained the most variance in cognitive stress symptoms, over and above all dimensions of IJDs. This means that when the main effects were examined, how the respondents emotionally experienced the current competence demands of the media industry was found to be more significant in terms of cognitive stress symptoms than the extent to which they experienced IJDs in their work. However, this does not necessarily challenge entirely the existence of a detrimental link between IJDs and cognitive stress symptoms: of the dimensions of IJDs, work intensification and intensified career-related planning and decision-making demands retained their positive association with cognitive stress symptoms, but the magnitude of this association depended on the level of the respondents’ competence demands-related negative affectivity and multitasking preference.

The findings highlight the importance of acknowledging underlying individual characteristics in seeking to understand the experience of work-related stress symptoms. More specifically, those for whom the current competence demands of the media industry elicited strong negative affects, such as tension and gloominess, reported overall more difficulties in cognitive functions at work (e.g., in concentrating and thinking clearly) than those who did not experience these negative affects. For those with strong negative affects, the higher the work intensification and intensified career-related planning and decision-making demands, the higher also the cognitive stress symptoms. However, for those with fewer competence demands-related negative affects, the level of cognitive stress symptoms was not dependent on the level of the IJD dimensions. These findings imply that on the one hand, a tendency to have negative mood states and to focus on the threatening aspects of one’s surroundings, such as the work environment, may be a risk factor that predisposes individuals to both IJDs and cognitive stress symptoms. On the other hand, the detrimental link between IJDs and cognitive stress symptoms might be alleviated if either competence demands or the negative affects related to those demands can be diminished.

The role of multitasking preference in the association between the IJD dimensions and cognitive stress symptoms was slightly different. Those low in preference to continually shift their attention from one thing to another did not, in general, suffer more from cognitive stress symptoms than those high in multitasking preference. This means that high or low multitasking preference did not function as an overall resource or risk factor, respectively, in relation to cognitive stress symptoms. However, multitasking preference played a role in the case of heightened experience of work intensification or intensified career-related planning and decision-making demands: among those more in favor of multitasking, these two IJD dimensions were not related to such high levels of cognitive stress symptoms as among those low in multitasking preference. These findings suggest that multitasking preference may to some degree protect against experiencing cognitive stress symptoms when IJDs are present, although it does not attenuate this detrimental association entirely.

The assumed protective role of competence demands-related positive affectivity was not detected, as in the regression models it was unrelated to the level of cognitive stress symptoms and did not moderate the association between IJDs and cognitive stress symptoms. However, as competence demands-related negative and positive affectivity were strongly and inversely related, the former may have explained some of the shared variance between low competence demands-related positive affectivity and high cognitive stress symptoms, although multicollinearity was not detected as a problem in the regression models. Another explanation might be that IJDs, especially work intensification and to some degree also intensified career-related planning and decision-making demands, are above all hindrance stressors, an explanation that supports earlier discussions about the challenge-hindrance nature of the different dimensions of IJDs (e.g., Podsakoff et al., 2007; Korunka et al., 2015; Paškvan et al., 2016; Mauno et al., 2019). Since individuals high in negative affectivity are sensitive to punishments (Carver and White, 1994) and threatening aspects of their work environment (e.g., Schaubroeck et al., 1992; Mäkikangas et al., 2013), the present results seem to suggest that, overall, IJDs trigger weaker reactions in those more inclined toward the inspiring and rewarding aspects of their work environment. In line with this interpretation, it has previously been shown that negative affectivity leads to increased risk perceptions, while the level of low or high positive affectivity has no such effect (Sobkow et al., 2016).

The cross-sectional design based on hierarchical moderated regression analyses used here does not confirm real, temporal cause-effect relations and allow causal interpretations, although it can be used for studying the moderating role of individual characteristics in *concurrent* stressor-stress reaction associations (Bolger and Zuckerman, 1995). Therefore, the results do not reveal whether IJDs really are stressors that induce cognitive stress symptoms, although we placed cognitive stress symptoms as dependent variable and IJDs as independent variables in our analyses. This was done based on job demands-resources theory (Demerouti et al., 2001; Bakker and Demerouti, 2017) and other studies that have presented IJDs as antecedents for occupational well-being (e.g., Korunka et al., 2015; Kubicek et al., 2015; Mauno et al., 2019).

Only 17% of the members of the Union of Journalists in Finland who opened the email inviting participation in the present study responded. Although low response rates are not uncommon when using web surveys (Blumenberg and Barros, 2018), this still raises questions about how far the present findings can be generalized. According to the information received by the research group from the Union of Journalists in Finland, 61% of their members are women and the mean age among members is about 45 years. In our sample these figures are 69% and 48 years, respectively, so it represents rather well the demographics of the target population. The Union of Journalists in Finland represents a heterogeneous group of media work professionals, who can be taken as good examples of knowledge workers working in a high-speed, high-pressure, and highly digitalized working environment (Malmelin and Villi, 2017; Westlund et al., 2020). We therefore believe that the present findings can be generalized to similar professions and industries in other countries. However, it is important that in future research the present tentative findings are replicated, that the phenomena are investigated in various occupational groups as well as with occupationally heterogeneous, representative samples of working age citizens across different cultures, and that information is gathered also from other informants and with other instruments than employee self-evaluation measures.

Although the present results suggest that competence demands-related negative affectivity may predispose individuals to cognitive stress symptoms in the presence of IJDs (here, work intensification and intensified career planning and decision-making demands), the present study design gives no information about the exact reasons for this. Negative affectivity is often associated with behavioral inhibition, a characteristic that represents the neurobiologically driven, behavioral tendency to avoid negative outcomes. A recent study shows that this avoidance tendency is also associated with higher sensitivity to the physiological signals arising from the autonomic nervous system (which form the essential component of stress reactions) (Lyyra and Parviainen, 2018). This could explain why negative affectivity may emphasize, and perhaps even strengthen, the experience of cognitive stress symptoms in conjunction with IJDs. Further studies are needed to reveal the dependencies and interactions between physiological sensitivity, affective tendencies, and experiences in cognitively demanding situations, to provide more individualized understanding, and to frame interventions to overcome the propensity to suffer stress at work.

## Implications and Conclusion

Altogether, the present findings support the view that when investigating and addressing the sources of stress at work, it is important to consider the dynamics between the individual and his/her working environment, that is, person-job fit (e.g., Kristof-Brown et al., 2005; Albertsen et al., 2010; van Vianen, 2018). It was shown here that both affective (competence demands-related negative affectivity) and motivational (multitasking preference) individual characteristics affected the strength of the relationship between IDJs and the cognitive stress symptoms experienced at work. This means that in addition to cognitive ergonomics workplace interventions (e.g., Kalakoski et al., 2020), also HR practices and interventions directed at easing employees’ negative affectivity related to competence demands at work, and at increasing multitasking ability and positive attitudes toward multitasking, may be fruitful.

These actions focusing on both the work environment and the ways in which employees perceive and encounter their work environment may support employees’ effective cognitive functioning even in the presence of IJDs that seem to have become a permanent part of contemporary working life (e.g., Obschonka et al., 2012; Kubicek et al., 2015). This idea is also in line with recent notions that for a more efficient HR management, it may be better to implement HR management in bundles rather than as isolated practices (for more see Salas-Vallina et al., 2020). Such actions could also support media and other knowledge workers in highly digitalized fields in maintaining their competence in crucial aspects of their work, such as creativity (Malmelin and Nivari-Lindström, 2017; Deuze, 2019) and knowledge absorption capacity (Salas-Vallina et al., 2020).

## Data Availability Statement

The raw data supporting the conclusions of this article will be made available by the authors, without undue reservation, provided that their requests do not violate the European Data Protection Regulation statutes.

## Ethics Statement

Ethical review and approval was not required for the study on human participants in accordance with the local legislation and institutional requirements. The patients/participants provided their written informed consent to participate in this study.

## Author Contributions

JR, PL, and TP conceived the study design. JR performed the statistical analysis and drafted the manuscript. PL, TF, and MV wrote sections of the manuscript and provided critical comments to it. All authors were involved in the data collection and approved the final version.

## Conflict of Interest

The authors declare that the research was conducted in the absence of any commercial or financial relationships that could be construed as a potential conflict of interest.
